# Active pattern on nailfold capillaroscopy in a patient with systemic sclerosis

**DOI:** 10.2478/rir-2023-0025

**Published:** 2023-09-27

**Authors:** Wenjing Ye, Ling Cao, Yingzi Zhou, Yu Xue, Weiguo Wan, Hejian Zou, Xiaoxia Zhu

**Affiliations:** Department of Rheumatology, Huashan Hospital, Fudan University, Shanghai, China; Institute of Rheumatology, Immunology and Allergy, Fudan University, Shanghai, China

A 65-year-old man presented with a two-year history of Raynaud’s phenomenon (RP) and a ten-month history of rapidly progress of skin swelling and hardening. Autoantibody testing revealed positivity for antinuclear antibodies (1: 1000) and anti-Scl70 antibodies, baseline serum creatine and blood pressure were normal. Nailfold capillaroscopy (NC) examination demonstrated decreased capillary density, along with enlarged (giant) capillaries, perivascular effusion, hemorrhage, and slowed blood flow (Figure 1A). Upon the diagnosis of diffuse cutaneous systemic sclerosis (SSc), the patient sought care at a local hospital, where he initiated treatment with prednisone (30 mg/d). However, he returned to our institution two weeks later, due to the possibility of a scleroderma renal crisis with weakness, elevated blood pressure, chest distress, nausea and increased serum creatinine.

**Figure 1:**
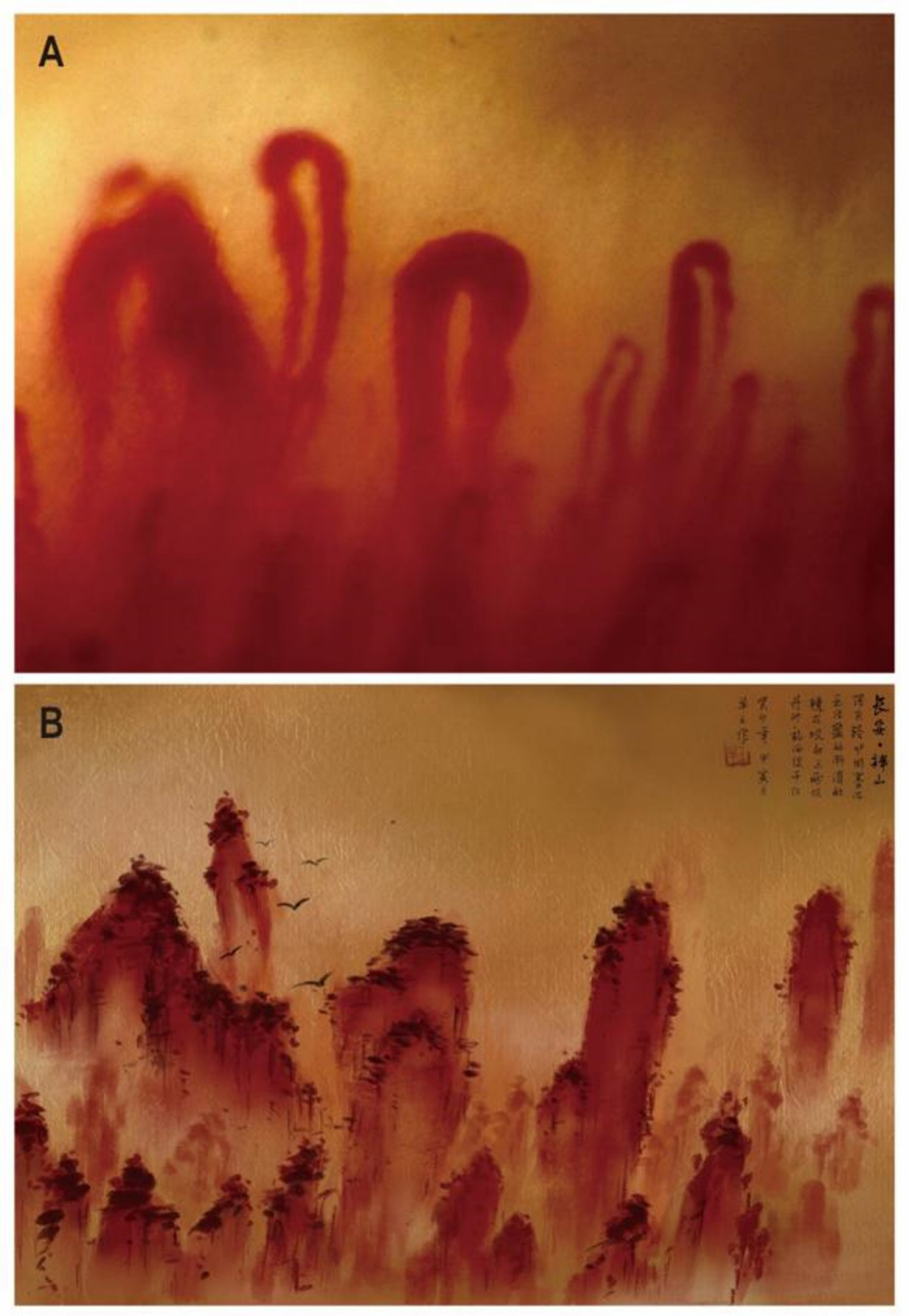
Nailfold capillary of the patient. (A) Nailfold capillaroscopy image of SSc patient; (B) Reproduction of (A) in traditional Chinese painting.

The aberrant blood capillaries observed in patients with SSc (Figure 1A), exhibit a striking resemblance to the intricate landscapes depicted in traditional Chinese paintings (Figure 1B). The capillaries intricately shape a myriad of mountain-like forms, and the perivascular effusion conjures an analogy to the veiling mist enveloping mountains, thereby intensifying the air of mystique. Furthermore, the hemorrhaging can be likened to the presence of dense bushes or trees adorning mountainous terrain.

As a non-invasive method, NC is a valuable way to predict the evolution of Rp and digital ulcers, facilitate SSc diagnosis, and offer insights into disease prognosis.^[1,2]^ In the context of our patient, the distinctive microvascular alterations observed by NC examination may light signals for kidney injury in the patient who suffered rapidly progression of skin sclerosis, and the subsequent administration of glucocorticoids may serve as a triggering factor for the onset of renal crisis. Consequently, in cases of SSc patients demonstrating rapidly involvement of skin, even displayed with anti-Scl70 positivity, the presence of characteristic microvascular damage might be predictive to the kidney crisis. Careful consideration should be given to the utilization of glucocorticoid therapy due to its potential implications.

## References

[1] Sulli A, Paolino S, Pizzorni C (2020). Progression of nailfold capillaroscopic patterns and correlation with organ involvement in systemic sclerosis: a 12 year study. Rheumatology (Oxford).

[2] van Leeuwen N, Ciaffi J, Schoones J (2021). The contribution of sex and auto-antibodies to microangiopathy assessed by nailfold videocapillaroscopy in systemic sclerosis: A systematic review of the literature. Arthritis Care Res (Hoboken).

